# Systemic-pulmonary collateral supply associated with clinical severity of chronic thromboembolic pulmonary hypertension: a study using intra-aortic computed tomography angiography

**DOI:** 10.1007/s00330-022-08768-6

**Published:** 2022-04-14

**Authors:** Wenyu Sun, Hideki Ota, Haruka Sato, Saori Yamamoto, Shunsuke Tatebe, Tatsuo Aoki, Koichiro Sugimura, Junya Tominaga, Hiroaki Shimokawa, Takuya Ueda, Kei Takase

**Affiliations:** 1 Department of Diagnostic Radiology, Tohoku University Graduate School of Medicine, 2-1, Seiryo-machi, Aoba-ku, Sendai, Miyagi 980-8575 Japan; 2 Department of Advanced MRI Collaboration Research, Tohoku University Graduate School of Medicine, 2-1, Seiryo-machi, Aoba-ku, Sendai, Miyagi 980-8575 Japan; 3 Department of Diagnostic Radiology, Tohoku University Hospital, 1-1, Seiryo-machi, Aoba-ku, Sendai, Miyagi 980-8574 Japan; 4 Department of Cardiovascular Medicine, Tohoku University Graduate School of Medicine, 2-1, Seiryo-machi, Aoba-ku, Sendai, Miyagi 980-8575 Japan; 5 Department of Cardiology, International University of Health and Welfare, Narita Hospital, 852, Hatakeda, Narita Chiba, 286-0124 Japan; 6 Graduate School, International University of Health and Welfare, School of Medicine, 4-3 Kozunomori, Narita Chiba, 286-8686 Japan; 7 Department of Clinical Imaging, Tohoku University Graduate School of Medicine, 2-1, Seiryo-machi, Aoba-ku, Sendai, Miyagi 980-8575 Japan

**Keywords:** Collateral circulation, CT angiography, Pulmonary hypertension

## Abstract

**Objectives:**

To assess whether systemic-pulmonary collaterals are associated with clinical severity and extent of pulmonary perfusion defects in chronic thromboembolic pulmonary hypertension (CTEPH).

**Methods:**

This prospective study was approved by a local ethics committee. Twenty-four patients diagnosed with inoperable CTEPH were enrolled between July 2014 and February 2017. Systemic-pulmonary collaterals were detected using pulmonary vascular enhancement on intra-aortic computed tomography (CT) angiography. The pulmonary enhancement parameters were calculated, including (1) Hounsfield unit differences (HUdiff) between pulmonary trunks and pulmonary arteries (PAs) or veins (PVs), namely HUdiff-PA and HUdiff-PV, on the segmental base; (2) the mean HUdiff-PA, mean HUdiff-PV, numbers of significantly enhanced PAs and PVs, on the patient base. Pulmonary perfusion defects were recorded and scored using the lung perfused blood volume (PBV) based on intravenous dual-energy CT (DECT) angiography. Pearson’s or Spearman’s correlation coefficients were used to evaluate correlations between the following: (1) segment-based intra-aortic CT and intravenous DECT parameters (2) patient-based intra-aortic CT parameters and clinical severity parameters or lung PBV scores. Statistical significance was set at *p* < 0.05.

**Results:**

Segmental HUdiff-PV was correlated with the segmental perfusion defect score (*r* = 0.45, *p* < 0.01). The mean HUdiff-PV was correlated with the mean pulmonary arterial pressure (PAP) (*r* = 0.52, *p* < 0.01), cardiac output (*rho* = − 0.41, *p* = 0.05), and lung PBV score (*rho* = 0.43, *p* = 0.04). And the number of significantly enhanced PVs was correlated with the mean PAP (*r* = 0.54, *p* < 0.01), pulmonary vascular resistance (*r* = 0.54, *p* < 0.01), and lung PBV score (*rho* = 0.50, *p* = 0.01).

**Conclusions:**

PV enhancement measured by intra-aortic CT angiography reflects clinical severity and pulmonary perfusion defects in CTEPH.

**Key Points:**

• *Intra-aortic CT angiography demonstrated heterogeneous enhancement within the pulmonary vasculature, showing collaterals from the systemic arteries to the pulmonary circulation in CTEPH.*

• *The degree of systemic-pulmonary collateral development was significantly correlated with the clinical severity of CTEPH and may be used to evaluate disease progression.*

• *The distribution of systemic-pulmonary collaterals is positively correlated with perfusion defects in the lung segments in CTEPH.*

## Introduction

Chronic thromboembolic pulmonary hypertension (CTEPH) is a severe progressive pulmonary disease, classified into the fourth group of pulmonary hypertension (PH) in the 2018 Nice classification [1, 2]. Without treatment, CTEPH can result in right ventricular failure and poor prognosis [3, 4].

**Fig. 1:**
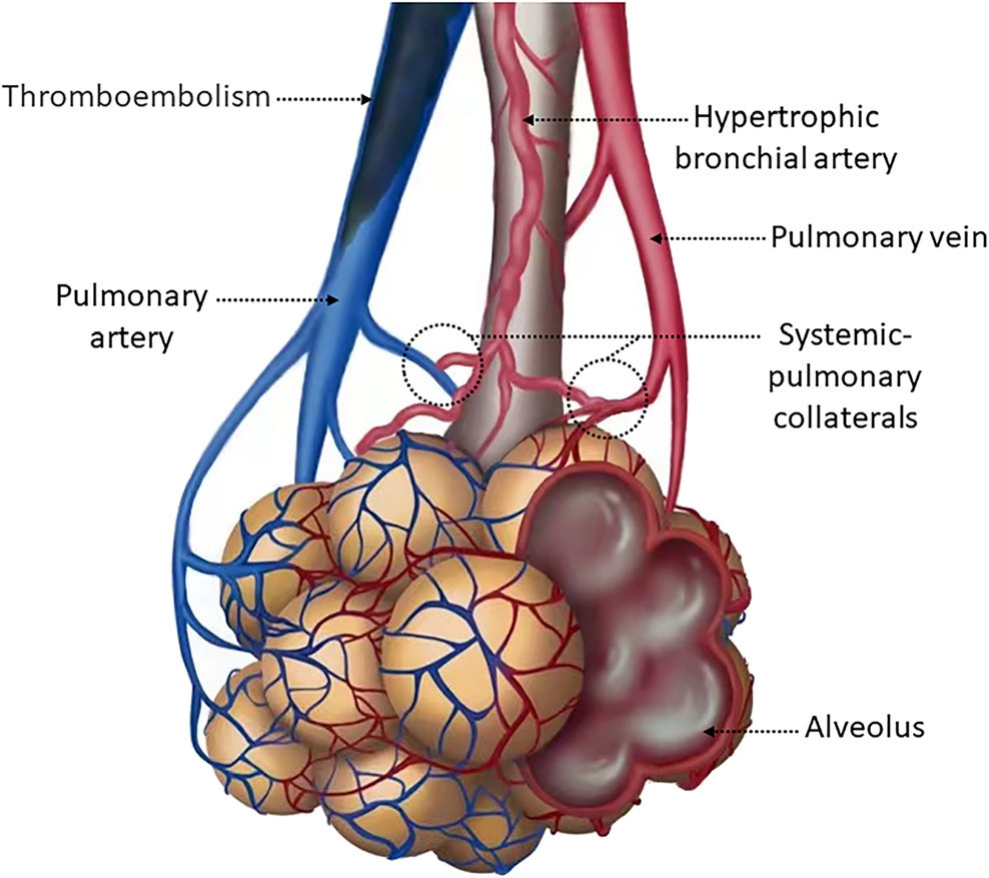
Schematic diagram of systemic-pulmonary collaterals on pulmonary vasculature in chronic thromboembolic pulmonary hypertension. Chronic thromboembolic material occludes the pulmonary artery. The bronchial arteries grew thicker with an increase in blood flow. Systemic collaterals are connected to both the pulmonary arteries and pulmonary veins

Systemic-pulmonary collaterals, a distinctive feature of CTEPH, may play an important role in its pathophysiology [4–7]. Due to persistent thrombus obstruction, pre-existing anastomoses between systemic and pulmonary circulation, which are functionally closed under normal conditions, might open and develop to supply blood to lung areas distal to occlusions [4, 8] (Fig. 1). These collateral supplies mainly come through dilated bronchial arteries [6, 9–12], the amount of which is 10–30 times larger than normal [7, 13]. Systemic-pulmonary collaterals may contribute to the development of small-vessel disease [6], which substantially affects PH progression, pulmonary vascular resistance (PVR), and postsurgical outcomes [4, 14, 15]. Studies reported that transcatheter occlusion of bronchopulmonary collaterals prior to pulmonary thromboendarterectomy (PEA) may reduce the incidence of reperfusion pulmonary oedema and improve early postoperative haemodynamic function [16, 17]. However, whether the degree of collateral flow contributes to the severity of CTEPH remains unclear. Radiological studies have not been performed to establish the relationship between the degree of systemic-pulmonary collateral flow and clinical severity in CTEPH. As it is secondary to thrombus embolism, the systemic-pulmonary collateral volume may differ among lung segments [6, 18]. However, the distribution of collateral flow into the lung vasculature and its relationship with the degree of pulmonary perfusion defects remains unclear.

Intra-aortic injection of contrast media prevents contamination of enhancements from the right heart system and can be used to detect and estimate bronchial-pulmonary shunt flow [7]. Enhancement of the pulmonary vasculature can purely reflect blood flow from systemic collaterals if the scan delay after injection initiation is minimised, allowing a quantitative evaluation of the shunt degree. Intravenously injected contrast-enhanced dual-energy computed tomography (DECT) can generate lung perfused blood volume (PBV) images that allow the detection of pulmonary perfusion defects [19–26]. However, there is a limitation in evaluating the degree of systemic-pulmonary shunts considering the dynamics of intravenously injected contrast media.

We aimed to evaluate the degree of shunt formation using intra-aortic CT angiography. We assessed whether the degree of contrast enhancement in the pulmonary vasculature, identified by CT angiography after intra-aortic injection, was associated with the clinical severity of CTEPH and pulmonary perfusion defects reflected in lung PBV images.

## Materials and methods

### Patients

**Fig. 2:**
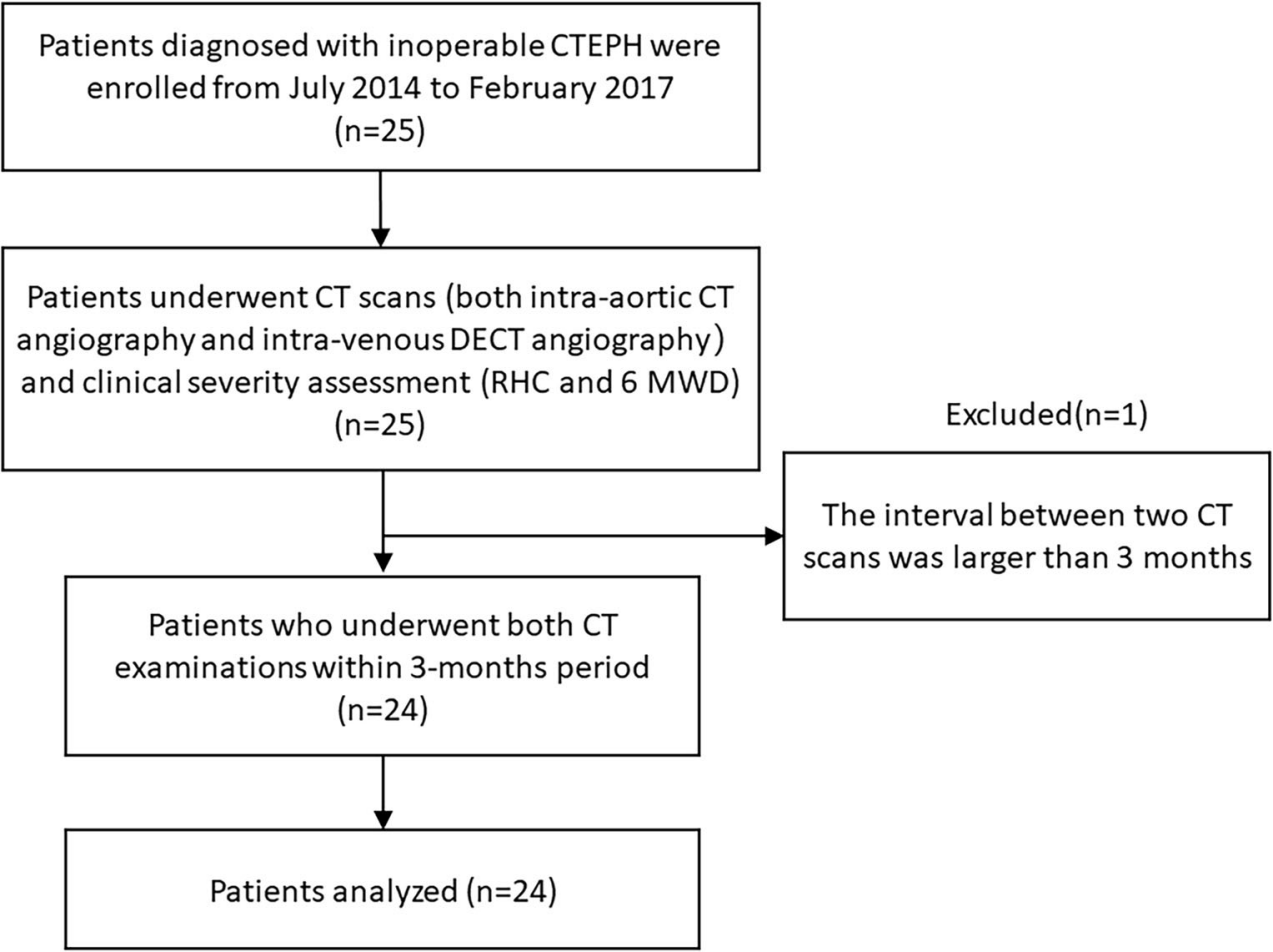
Flowchart of patients’ enrolment. Patients were diagnosed with chronic thromboembolic pulmonary hypertension by experienced cardiologists based on the standard diagnostic criteria, including lasting symptoms for more than six months, precapillary pulmonary hypertension by right heart catheterisation (RHC), segmental perfusion defects on ventilation/perfusion (V/Q) scintigraphy, and typical angiography findings. Patients were considered inoperable based on the distribution of disease (peripheral type) as well as their general conditions. All patients were already in chronic condition at enrolment, and no surgical or endovascular intervention was performed within the 3-month interval between the two CT scans. 6 MWD, six-min walking distance

This prospective study was approved by the local ethics committee; written informed consent was obtained from all patients. Twenty-five consecutive patients (3 men, 22 women; mean age: men, 62 years [range, 48–77]/women 69 years [range, 46–83]) diagnosed with inoperable CTEPH based on the current standard criteria between July 2014 and February 2017 were enrolled [1] (Fig. 2). All patients underwent left- and right-sided heart catheter examinations as part of the CTEPH work-up to determine their treatment strategy; during left-sided heart catheter examinations, intra-aortic CT angiography was performed to detect systemic-pulmonary collateral supply. All patients also underwent intravenous DECT angiography. Patients who underwent both CT examinations within 3 months period, assuming no changes in clinical conditions under the management of CTEPH, were included (Fig. 2).

Sample size was calculated as previously reported [13]. The total sample size required was 19, with a moderate setting (*α* = 0.05, *β* = 0.20, *r* = 0.60) for the correlation.

### Intra-aortic CT angiography image acquisition

Intra-aortic CT angiography was performed using one of two multidetector CT scanners (SOMATOM Definition Flash, Siemens Healthineers, or Aquilion ONE ViSION Edition, Canon Medical Systems). An intra-aortic injection into the ascending aorta was performed using a 4-Fr pigtail catheter. A total of 105 ml of 1:3 diluted iodine contrast media (35 ml of 350 mg I/ml contrast media diluted with 70 ml of saline) was injected at a rate of 9 ml/s using a power injector [27, 28]. CT imaging was performed with a scan delay of 9 s and included the entire chest during a single breath-hold in the head-to-foot direction. Other imaging parameters are listed in Table 1. Heart rate was recorded during acquisition.

**Table 1:** Imaging parameters of intra-aortic CT angiography

Scanner	Aquilion ONE ViSION Edition (Canon Medial Systems)	SOMATOM Definition Flash (Siemens Healthineers)
Tube voltage	80 kVp	80 kVp
Collimation	0.5 mm × 100 raw	0.6 mm × 64 raw
Slice thickness/interval (mm)	1/1	1/1
Pitch factor	0.81	1.0
Gantry rotation speed (s/rotation)	0.275	0.28
Tube current	Automatic modulation	Automatic modulation
Reconstruction kernel	Soft tissue	Soft tissue

### Intra-aortic CT angiography image analysis

**Fig. 3:**
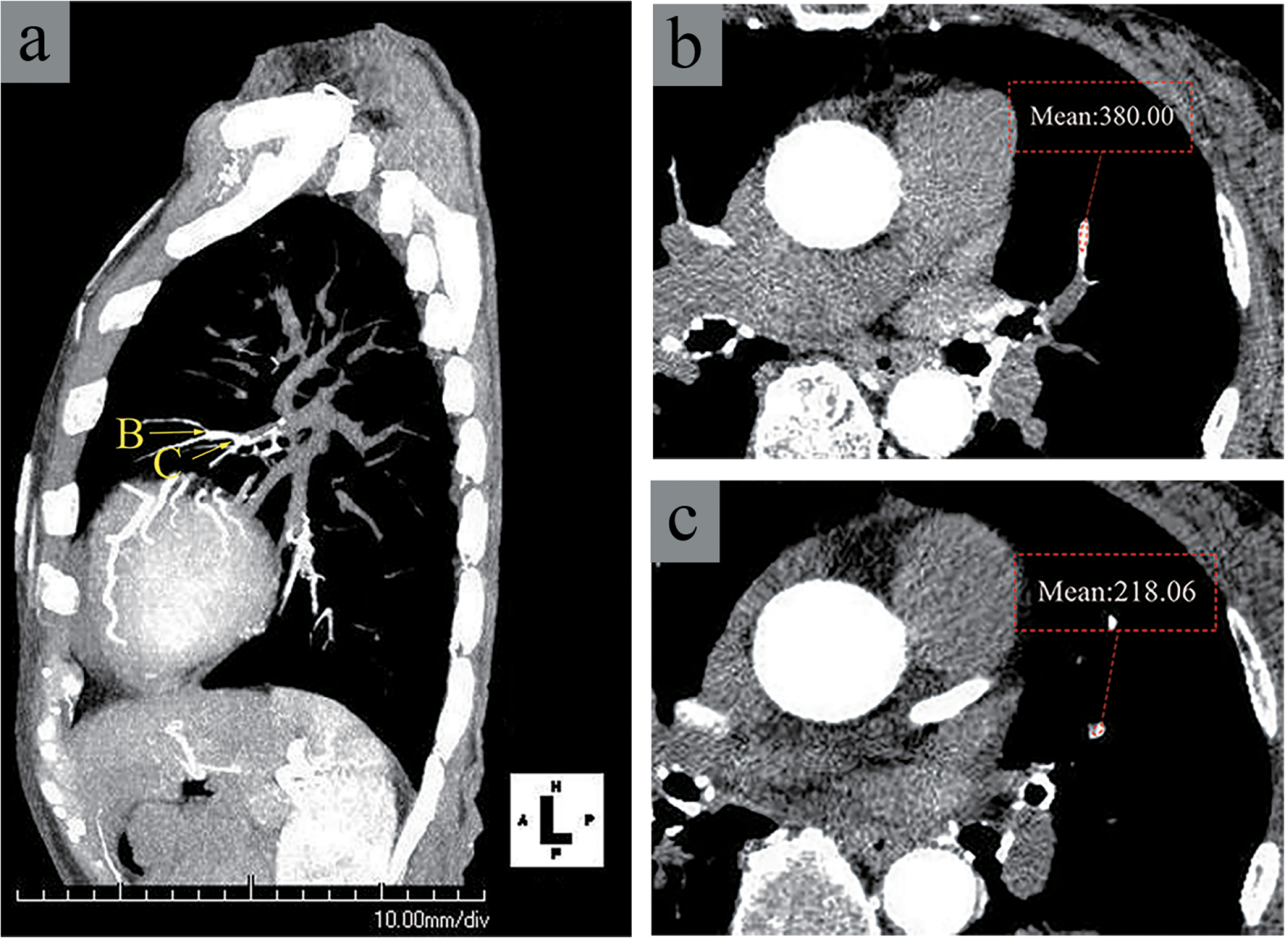
Enhanced pulmonary arteries shown on intra-aortic computer tomographic angiography in a representative patient (72-year-old woman with chronic thromboembolic pulmonary hypertension). **a** Sagittal view of the partial maximum projection (MIP) image with 20 mm slice thickness showing enhancement in the pulmonary artery of the left S4 by contrast media injected in the ascending aorta; arrows B and C indicate slice levels of the corresponding original transverse images shown in **b** and **c**, respectively. Transverse images at levels corresponding to arrows B and C in the image shown in **a**. The numbers indicate the mean CT values (HU) at regions of interest (ROIs) placed on the segmental pulmonary artery (A4). The mean of the CT values was regarded as the HU-PA for the left A4

**Fig. 4:**
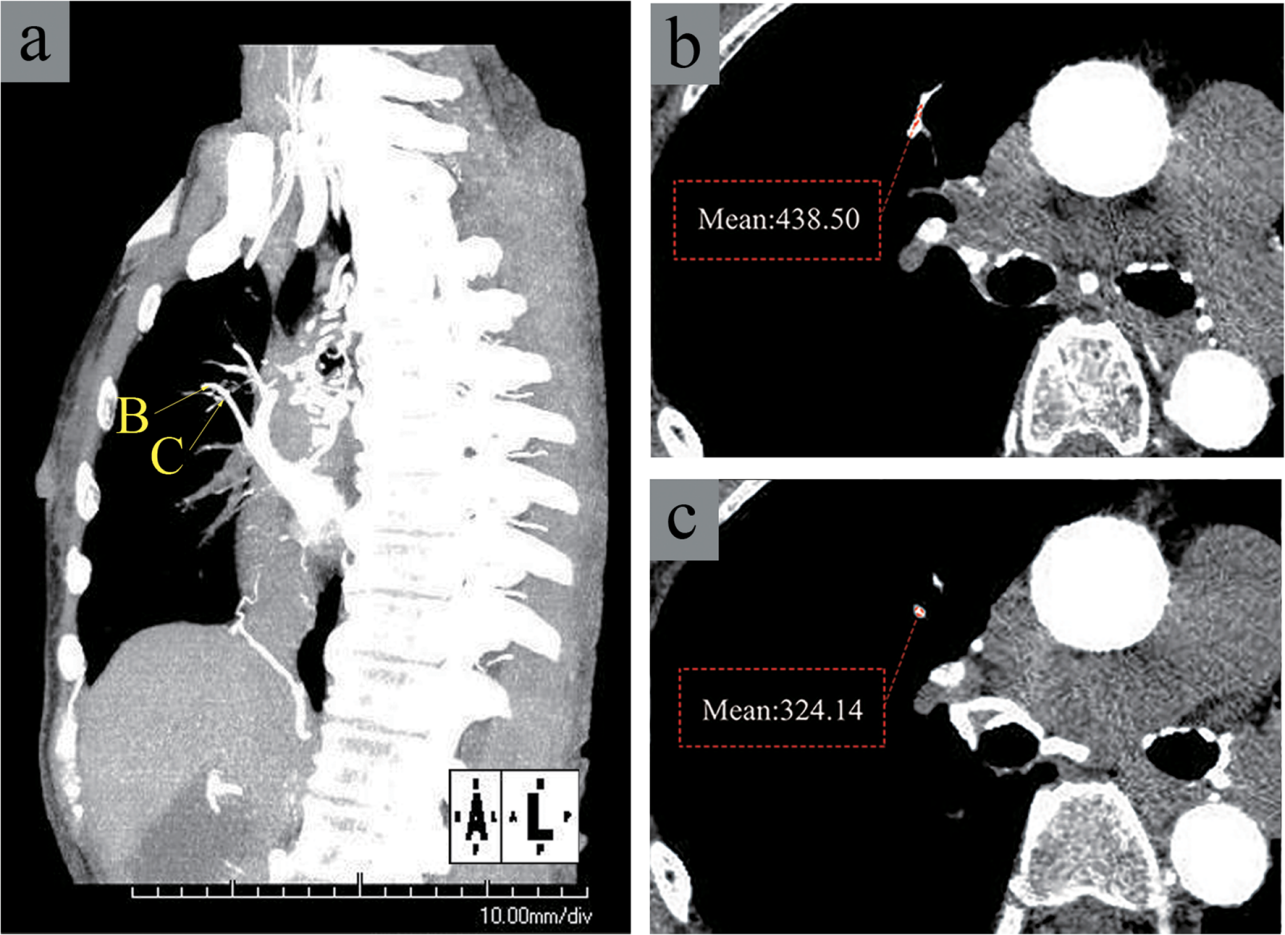
Enhanced pulmonary veins shown on intra-aortic computer tomographic angiography in a representative patient (72-year-old woman with chronic thromboembolic pulmonary hypertension). **a** Left anterior oblique view (60 degrees) MIP image with 20 mm slice thickness showing enhancement in the pulmonary veins of the right S3; arrows B and C indicate slice levels of the corresponding original transverse images shown in **b** and **c**, respectively. Transverse images at the levels corresponding to arrows B and C, respectively, in the image shown in **a**. The numbers indicate mean CT values (HU) of ROIs placed on the segmental pulmonary vein (V3). The mean of the mean CT values was regarded as the HU-PV for the right V3

Two radiologists, blinded to patient clinical information, evaluated the CT images through visual observations performed with a window width of 350 Hounsfield units (HU) and a window level of 75 HU. Circular regions of interest (ROIs) were placed by consensus where enhancement appeared in the individual vessels on axial images. Multiple ROIs (3–5) were placed across multiple sections of each vessel. The mean CT values of the two highest-enhanced ROIs for each vessel were selected to represent the degree of vascular enhancement in the corresponding lung segment (Figs. 3 and 4). Measurements were recorded separately for the pulmonary arteries (PAs) and pulmonary veins (PVs) in 18 lung segments as HU-PAs and HU-PVs. The CT value of the ROI on the pulmonary trunk (PT) was recorded as HU-PT for each patient. One reviewer performed second measurements of HU-PAs, HU-PVs, and HU-PT to evaluate the intraobserver agreement.

### Vessel-based assessment for the degree of pulmonary vascular enhancement

Considering the haemodynamics of contrast media injected from the ascending aorta at a 9-s scan delay, we assumed that no contrast medium entered the PT. Therefore, we used the HU-PT value as a reference for the non-enhanced intraluminal blood density. We then defined the degree of contrast enhancement in the target segmental vessel as the Hounsfield unit difference (HUdiff), calculated as HU-PA, HU-PV, minus HU-PT. For each patient, the HUdiff values for PAs and PVs were recorded separately for 18 lung segments as HUdiff-PAs and HUdiff-PVs, to represent the degrees of systemic-pulmonary collateral development observed in the PAs or PVs.

We recorded the Hounsfield unit standard deviation (SD) of the ROI on the PT for each patient to evaluate the significant enhancement [29]. Significantly enhanced PAs and PVs were defined separately as the corresponding HUdiff-PA or HUdiff-PV greater than twice the Hounsfield unit SD of the PT and recorded for all patients.

### Patient-based assessment of the degree of pulmonary vascular enhancement

For patient-based analysis, the mean HUdiff-PA and mean HUdiff-PV were calculated as the average values of 18 segmental HUdiff-PA or HUdiff-PV separately for each patient.

The total number of significantly enhanced PAs and PVs were calculated separately for each patient.

### Lung PBV image acquisition

Intravenous DECT angiography was performed within 3 months of intra-aortic CT angiography (Fig. 2) using a second-generation dual-source CT scanner (SOMATOM Definition Flash, Siemens Healthineers). All patients were scanned in the pulmonary arterial phase to minimise the influence of systemic-pulmonary collaterals. Detailed scanning protocols are summarised in Table 2.

**Table 2:** Acquisition and injection parameters for intravenous dual-energy CT angiography examinations

	Dual-source CT scanner
Tube A	80 kv–141 mAs
Tube B	140 Sn–60 mAs
Collimation	64 × 0.5 mm × 2
Pitch	1
Rotation time, s	0.28
Acquisition	Caudocranial
Iodine concentration, mg I/ml	350
Flow rate, ml/s/kg	0.075
ROI position	Main pulmonary artery

### Lung PBV image evaluation

**Fig. 5:**
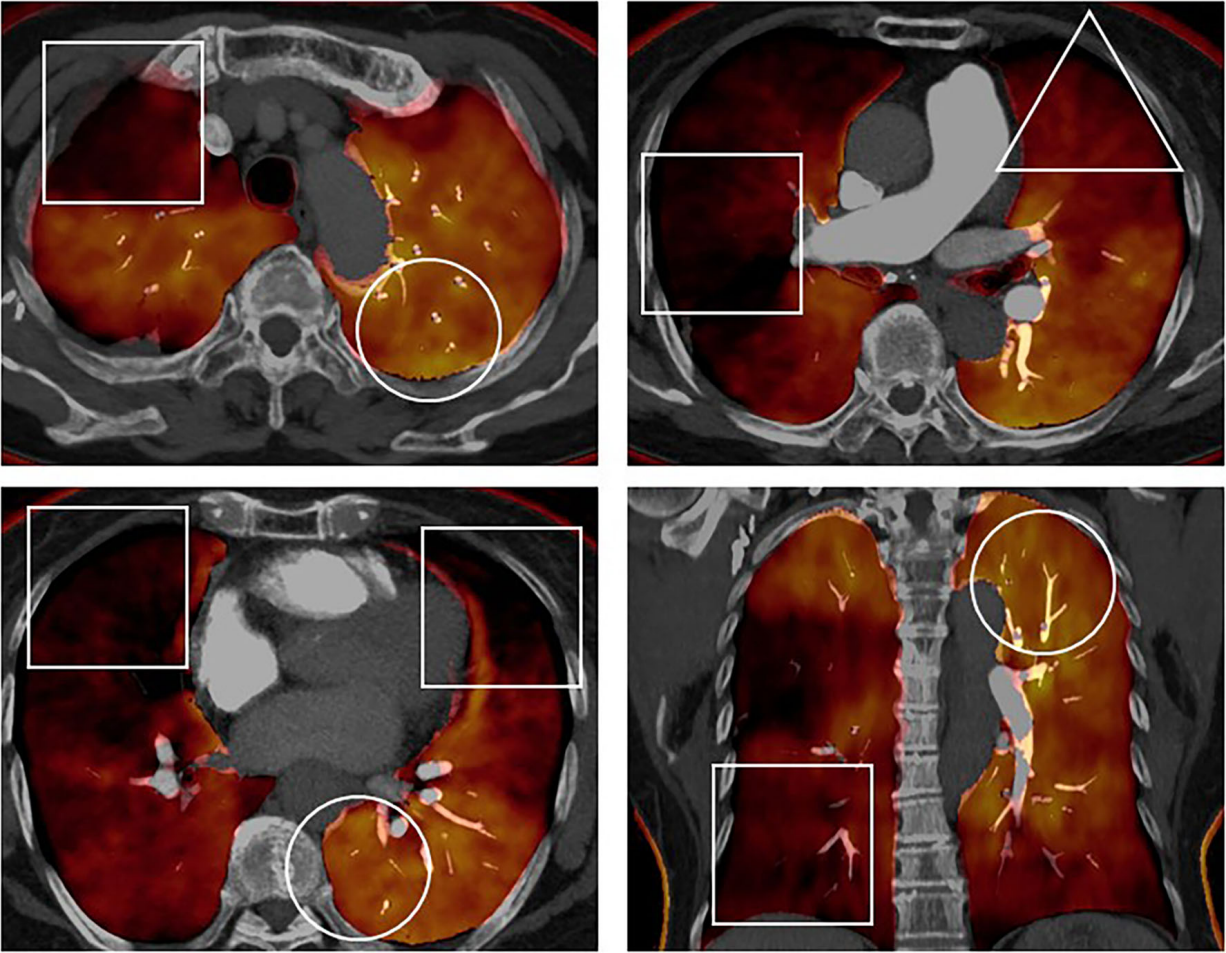
Evaluation of lung perfused blood volume (PBV) image in a representative patient. (76-year-old woman with chronic thromboembolic pulmonary hypertension). On these images, areas that were black or dark orange were considered hypoperfused, and areas that were bright orange or yellow were normo-perfused. No-perfusion defect segments (score 0) were reflected using circles, a less-than-half perfusion defect segment (score 1) was reflected using triangles, and a more-than-half perfusion defect segment (score 2) was reflected using tetragons

The same two radiologists, blinded to the patients’ demographic and clinical information, scored the extent of pulmonary perfusion defects by consensus using both axial and coronal colour-coded lung PBV images. The scoring standards for lung PBV images suggested by Takagi et al were applied (3-point scale: 0, no defect; 1, defect < half segmental volume; 2, defect > half segmental volume) [20] (Fig. 5). The perfusion defect score of each segment was recorded for all patients. The final lung PBV score was calculated as the sum of the perfusion defect scores of the 18 lung segments for each patient.

### Assessment of the laterality of pulmonary vascular enhancement and pulmonary perfusion defect

We compared the laterality between the right and left lung for pulmonary vascular enhancement parameters and pulmonary perfusion defects separately as follows: the mean of HUdiff-PA and HUdiff-PV, the percentage of significantly enhanced PAs and PVs, and the mean perfusion defect scores of the 10-right lung segments versus those of the 8-left lung segments.

### Assessment of clinical severity

All patients underwent right heart catheterisation (RHC) during the same session as the intra-aortic CT angiography examination. Pulmonary arterial pressure (PAP) (systolic, diastolic, and mean), PVR, right atrial pressure (RAP), pulmonary arterial wedge pressure (PAWP), cardiac index (CI), and cardiac output (CO) values were obtained. Brain natriuretic peptide (BNP) levels and 6-min walking distance (6 MWD) were measured within 3 months of intra-aortic CT angiography. No surgical or endovascular interventions were performed during this period.

### Statistical analysis

Descriptive statistics were presented as means with SDs for normally distributed variables, medians with interquartile ranges for non-normally distributed variables, and numbers of cases (and percentages) per group for categorical variables. Sex differences in pulmonary vascular enhancement and pulmonary perfusion defect parameters were assessed separately using an independent *t-*test. The HU-PT values, as the reference, were compared between the two groups imaged with different scanners using an independent *t-*test. Within-subject laterality was evaluated using paired *t*-test. Intraobserver agreement of pulmonary vascular enhancement data was determined using the intraclass correlation coefficient (ICC). Correlations between the findings of intra-aortic CT angiography, RHC, other clinical parameters, and lung PBV imaging findings were evaluated using Pearson’s or Spearman’s correlation coefficients. To accommodate the use of multiple variables per patient, the standard errors of the variables were estimated using bootstrapping methods with 10,000 samples. Multivariable linear regression analyses were used to assess whether different scanners were confounding factors in the correlation analysis. Statistical computations were performed using IBM SPSS Statistics for Windows (version 21.0. IBM Corp.). Statistical significance was set at *p* < 0.05.

## Results

One patient was excluded from this study because the interval between the two scans was over three months. Consequently, 24 patients were included in the final analysis (Fig. 2).

### RHC examination and other clinical severity assessment

The mean ± SD of RHC parameters, BNP of 24 patients, and 6 MWD for 21 patients were recorded; the remaining three patients were in poor physical condition and could not undergo the 6 MWD test (Table 3).

**Table 3:** Summary of the patient’s clinical severity parameters

sPAP	73.29 ± 17.86 mmHg [range, 40–100].
dPAP	24.25 ± 7.95 mmHg [range, 13–41].
mPAP	41.21 ± 9.63 mmHg [range, 23–58].
PVR	851.33 ± 355.11 dyne s cm^−5^ [range, 269–1600]
PAWP	7.58 ± 2.95 mmHg [range, 2–14]
RAP	4.33 ± 2.10 mmHg [range, 1–9]
CO	2.97 ± 0.73 l/min [range, 1.6–4.14]
CI	2.05 ± 0.49 l/min/m^2^ [range, 1.1–3.0]
BNP	194.99 ± 210.15 pg/ml [range, 10.0–609.4]
6 MWD (21 patients)	371.14 ± 65.61 m [range, 265–488]

Note. Values are mean ± SD. *sPAP*, systolic pulmonary artery pressure; *dPAP*, diastolic pulmonary artery pressure; *mPAP*, mean pulmonary artery pressure; *PVR*, pulmonary vascular resistance; *PAWP*, pulmonary arterial wedge pressure; *RAP*, right atrial pressure; *CO*, cardiac output; *CI*, cardiac index; *BNP*, brain natriuretic peptide, *6 MWD*, 6-min walking distance. Three patients did not undergo the 6 MWD test because of their poor physical condition

### Intra-aortic CT angiography

All intra-aortic CT angiographies were successfully performed, without adverse events. The mean ± SD of the CT dose index for the 24 patients was 5.6 ± 3.5 mGy.

### Vessel-based assessment for the degree of pulmonary vascular enhancement

Intraobserver agreements of both PA and PV enhancement parameters evaluated on intra-aortic angiography images were excellent (ICC > 0.95). Image data were collected from all 24 patients as a total of 432 HU-PAs (mean, 120.4 ± 76.2 HU; range, 26.5–526.0 HU), 432 HU-PVs (mean, 134.9 ± 72.8 HU; range, 20.0 – 408.0 HU), and 24 HU-PTs (mean, 62.1 ± 17.3 HU; range, 43.5–103.3 HU); they were calculated into 432 HUdiff-PAs (mean, 59.3 ± 73.9 HU; range, 0 – 471.2 HU) and 432 HUdiff-PVs (mean, 73.6 ± 70.5 HU; range, 0–356.2 HU). A total of 176 and 224 PAs and PVs were significantly enhanced, respectively, in 24 patients.

### Patient-based assessment for the degree of pulmonary vascular enhancement

We calculated the mean HUdiff-PAs (mean, 59.3 ± 38.7 HU; range, 6.8–172.8 HU) and mean HUdiff-PVs (mean, 73.6 ± 36.3 HU; range, 23.0–181.0 HU) on the patient base. We also calculated the total number of significantly enhanced PAs (median, 7; range, 0–14) and PVs (median, 10; range, 3–15) on the patient base. Patients’ heart rates during image acquisition were not correlated with mean HUdiff-PA (*r* = 0.23, *p* = 0.28) or mean HUdiff-PV (*r* = − 0.01, *p* = 0.96). No sex differences were observed in the aforementioned parameters.

### Pulmonary perfusion defect assessment

We evaluated 432 perfusion defect scores for all pulmonary segments (median, 1; range, 0–2) and calculated lung PBV scores (median, 23; range, 16–31) for 24 patients. No sex differences were observed in the aforementioned parameters.

### Correlation between pulmonary vascular enhancement parameters and the clinical severity of CTEPH

**Fig. 6:**
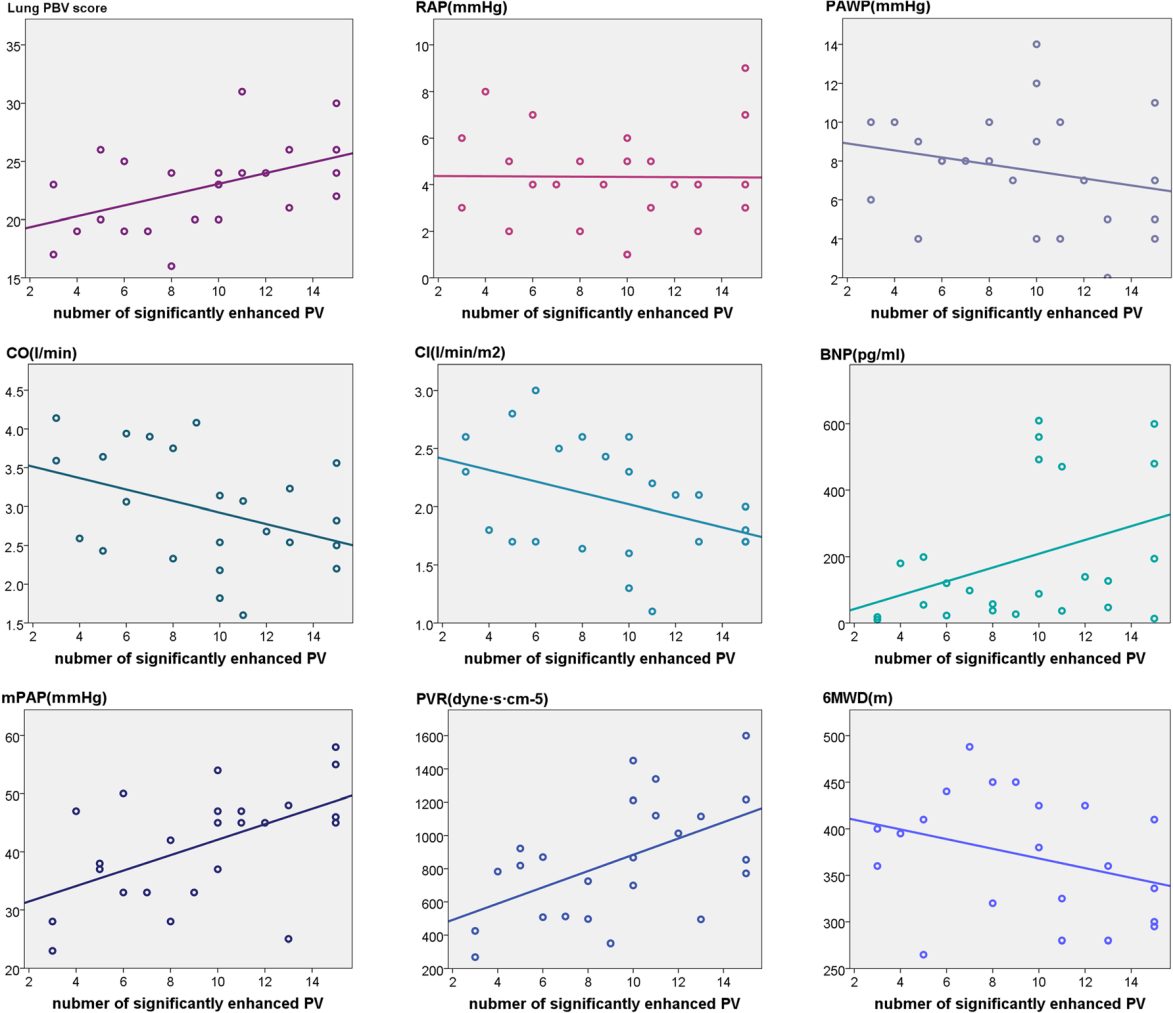
Correlations between the total number of significantly enhanced PVs and clinical severity parameters, as well as lung perfused blood volume scores (PBV) in patients with CTEPH. Positive correlations were found between the total number of significantly enhanced PVs and the mean PAP (mPAP), PVR, and lung PBV scores in patients with CTEPH. Marginally negative correlations were observed between the number of significantly enhanced PVs and CO and CI (*p* < 0.01 for all comparisons). No significant correlations were found for any of the other parameters

We compared patient-based pulmonary vascular enhancement parameters with clinical severity parameters by using Pearson’s and Spearman’s correlation coefficients. PV enhancement parameters were correlated with several clinical severity parameters. Significant correlations were found between the mean HUdiff-PV and mean PAP, systolic PAP, and CO; the total number of significantly enhanced PVs and mean PAP, systolic PAP, and PVR (Fig. 6). The total number of significantly enhanced PVs also showed a marginal association with CO and CI (Fig. 6). The details are summarised in Table 4. No significant correlations were found between PA enhancement and clinical severity parameters.

**Table 4:** Summary of the correlation between the enhancement parameters of pulmonary vein and clinical severity

	Mean HUdiff-PV		Number of significantly enhanced PVs	
	*r*	*p*	*r*	*p*
sPAP	0.46	0.02 *	0.51	0.01 *
dPAP	0.27	0.20	0.36	0.09
mPAP	0.52	< 0.01*	0.54	< 0.01*
PVR	0.28	0.19	0.54	< 0.01*
PAWP	− 0.04	0.86	− 0.24	0.26
RAP	0.10	0.63	− 0.01	0.97
CO	− 0.41	0.05*	− 0.39	0.06
CI	− 0.27	0.20	− 0.38	0.06
BNP	0.22	0.30	0.36	0.09
6 MWD	− 0.27	0.24	− 0.26	0.26

Note. Values are mean ± SD. *HUdiff*, Hounsfield unit difference; *PV*, pulmonary vein; *sPAP*, systolic pulmonary artery pressure; *dPAP*, diastolic pulmonary artery pressure; *mPAP*, mean pulmonary artery pressure; *PVR*, pulmonary vascular resistance; *PAWP*, pulmonary arterial wedge pressure; *RAP*, right atrial pressure; *CO*, cardiac output; *CI*, cardiac index; *BNP*, brain natriuretic peptide; *6 MWD*, 6-min walking distance. Three patients skipped the 6 MWD test because of their poor physical condition. Correlations of CT angiography parameters with CO and CI were evaluated using Spearman’s correlation coefficient, while the rest were evaluated using Pearson’s correlation coefficient

### Correlation between pulmonary vascular enhancement parameters and the pulmonary perfusion defects

We performed lung segment-based and patient-based evaluations to study the relationship between pulmonary vascular enhancement parameters and pulmonary perfusion defect parameters using Spearman’s correlation. In the lung segment-based evaluation, a positive correlation was found between the HUdiff-PV and perfusion defect scores (*r* = 0.45, *p* < 0.01). No correlation was found between the HUdiff-PA and perfusion defect scores (*r* = 0.06, *p = *0.22).

In the patient-based evaluation, a positive correlation was found between the mean HUdiff-PV and lung PBV scores (*r* = 0.43, *p* = 0.04). A positive correlation was also found between the total number of significantly enhanced PVs and lung PBV score (*r* = 0.50, *p* = 0.01) (Fig. 6). No correlation was found between PA enhancement parameters and lung PBV score.

### Subgroup analysis based on the CT scanner used

The Siemens scanner was used in 16 patients (mean HU-PT, 65.3 ± 15.7 HU), and the Canon scanner was used in 8 patients (mean HU-PT, 55.8 ± 19.5 HU). The HU-PT values were not significantly different between the two groups (*p* = 0.21). In multivariable linear regression analyses, the scanners were not identified as significant confounding factors (*p* = 0.28 – 0.59) in the assessment of correlations between pulmonary enhancement parameters and clinical severity parameters or perfusion defect scores.

### Laterality of pulmonary vascular enhancement and pulmonary perfusion defects

Right-sided dominance was found for the mean of HUdiff-PA and mean of HUdiff-PV, the percentage of significantly enhanced PAs and PVs, and the mean perfusion defect scores of 24 patients. The details are summarised in Table 5.

**Table 5:** Laterality of pulmonary vascular enhancement and pulmonary perfusion defect

	*Right lung*	*Left lung*	*p*
Mean of HUdiff-PA (HU)	66.57 ± 45.51	50.29 ± 35.52	0.01
Mean of HUdiff-PV (HU)	85.45 ± 45.43	58.79 ± 34.53	< 0.01
Percentage of significantly enhanced PA	0.46 ± 0.26	0.34 ± 0.18	0.02
Percentage of significantly enhanced PV	0.59 ± 0.22	0.43 ± 0.30	0.02
Mean pulmonary perfusion defect scores	1.41 ± 0.25	1.08 ± 0.36	< 0.01

Note. Values are mean ± SD. *HUdiff*, Hounsfield unit difference; *PA*, pulmonary artery; *PV*, pulmonary vein

## Discussion

Using intra-aortic CT angiography to reflect systemic-pulmonary collaterals in CTEPH, we evaluated the degree of contrast enhancement in each lung segment of the PA and PV. We then assessed the relationship between the degree of shunt enhancement and pulmonary perfusion defects, as well as clinical severity parameters. This study has several major findings. First, in patients with CTEPH, the PV enhancement degree correlated positively with the mean PAP (patient-based), and the total number of PVs with significant enhancement correlated with important clinical severity parameters, including mean PAP and PVR (patient-based). Second, the degree of enhancement in the PV correlated positively with the degree of pulmonary perfusion defects (lung segment-based), and the overall PV enhancement degree correlated positively with the lung PBV score (patient-based). We also found that pulmonary vascular enhancement and pulmonary perfusion defects showed right-sided dominance in the CTEPH group. The observed enhancement in the pulmonary vasculature reflected the first-pass iodine contrast. The contrast medium coursed through a systemic-pulmonary shunt into the PVs. Therefore, the degree of enhancement reflects the degree of systemic collateral development.

Our study is the first to assess the detailed segmental distribution of systemic-pulmonary collaterals and verify their value in reflecting CTEPH severity. The positive correlation between the degree of systemic collateral flow and mean PAP and PVR suggests that shunts may play an important role in CTEPH progression. Exposure to high-pressure systemic circulation may induce small-vessel disease, contributing to increased PH and PVR [4, 6]. Studies have reported that the reduction in systemic collateral supply by transcatheter embolization results in a positive postoperative outcome, with reduced reperfusion pulmonary oedema [16, 17]. Furthermore, a recent study found that patients with higher PV small-vessel disease in the occluded lung area tend to have a worse prognosis or higher mortality after surgery [15]. Their results are consistent with our findings, as collaterals primarily perfuse into distal occluded lung areas and are the major contributor to small-vessel disease in this area [6]. As such, the degree of collateral vessels reflected by intra-aortic CT angiography may have potential value in preoperative planning and treatment outcome prediction. It is unknown whether systemic collaterals play a role in the treatment effect of local therapy, especially balloon pulmonary angioplasty (BPA). Further studies are warranted to evaluate the potential associations between the treatment effects of BPA and the presence of collaterals in targeted segmental arteries visualised by intra-aortic CT angiography. Our study is the first to reveal that a higher degree of systemic shunt is associated with more severe pulmonary arterial occlusion and pulmonary perfusion defects. It is not surprising that the more severe the ischaemia, the greater the collateral circulation required. Systemic collaterals may maintain airway epithelial oxygenation in the occluded lung areas [9, 30]. The shunt enhancement parameters obtained from the PV were more strongly correlated with the mean PAP than those obtained from the PA. We hypothesised that CT could not visualise pulmonary arterial enhancement distal to the anastomosis from the systemic arterial collaterals; isolated arterial anastomoses had an inner diameter of 50–150 μm [31], which was beyond the spatial resolution of CT. However, when shunt flow drains into the pulmonary venous system, contrast media passing through the shunt may be observed on CT. Additionally, anastomoses not only develop between the systemic circulation and PAs but also with PVs, and a pathological study reported that marked post-capillary remodelling and bronchial-artery-to-PV shunting were present in patients with CTEPH [6] (Fig. 1).

Our study has several limitations. First is the small sample size. Larger studies are required to validate our results. Second, we only assessed the degree of systemic collateral development before CTEPH treatment. Comparisons between changes in the systemic collaterals and clinical severity of CTEPH and changes in pulmonary perfusion and other treatment outcomes are necessary to confirm the clinical value of our study. Third, the time interval between the two CT examinations was not minimised in all patients. However, only chronic patients were enrolled, and their clinical conditions did not change under close management with anticoagulant drugs and vasodilators. Fourth, intra-aortic CT angiography is a feasible technique for assessing systemic-pulmonary collaterals; however, because of its invasiveness, indications should be carefully evaluated by a multidisciplinary CTEPH team in routine clinical settings. Fifth, our results are based on manual measurements. In the future, an alternative automated quantitative tool may be required to improve the reliability of shunt measurements and reduce bias and time consumption caused by manual measurements.

In conclusion, intra-aortic CT angiography demonstrated heterogeneous enhancement, reflecting collateral circulation from the systemic arteries to the pulmonary circulation in CTEPH. The mean HUdiff-PV and number of significantly enhanced PVs may help estimate the clinical severity of CTEPH and reflect the extent of pulmonary perfusion defects in patients. Our findings provide a better understanding of haemodynamic changes in CTEPH and provide insight into the management of patients with CTEPH.

## Abbreviations

BPA: Balloon pulmonary angioplasty
BNP: Brain natriuretic peptide
CI: Cardiac index
CO: Cardiac output
CT: Computed tomography
CTEPH: Chronic thromboembolic pulmonary hypertension
DECT: Dual-energy CT
HUdiff: Hounsfield unit difference
PA: Pulmonary artery
PAP: Pulmonary arterial pressure
PAWP: Pulmonary arterial wedge pressure
PBV: Perfused blood volume
PEA: Pulmonary endarterectomy
PT: Pulmonary trunk
PV: Pulmonary vein
PVR: Pulmonary vascular resistance
RAP: Right atrial pressure
ROIs: Regions of interest
SD: Standard deviation
6 MWD: 6-min walking distance

## Declarations

### Guarantor

The scientific guarantor of this publication is Hideki Ota; Department of Diagnostic Radiology, Tohoku University Hospital.

### Statistics and biometry

No complex statistical methods were necessary for this paper.

### Methodology

• prospective

• diagnostic or prognostic study

• performed at one institution
